# *Camptostemon philippinensis*, a new record of endangered mangrove species in the Balikpapan Bay, East Kalimantan, Indonesia

**DOI:** 10.12688/f1000research.140887.1

**Published:** 2023-10-23

**Authors:** Bina Swasta Sitepu, Abdul Razaq Chasani, Mukhlisi Mukhlisi, Tri Atmoko, Burhanuddin Adman, Istiana Prihatini

**Affiliations:** 1 Research Center for Ecology and Etnobiology, National Research and Innovation Agency, Bogor, West Java, 16911, Indonesia; 2 Faculty of Biology, Universitas Gadjah Mada, Yogyakarta, Special Region of Yogyakarta, 55281, Indonesia; 3 Research Center for Applied Zoology, National Research and Innovation Agency, Bogor, West Java, 16911, Indonesia; 4 Research Center for Plant Conservation, Botanic Gardens, and Forestry, National Research and Innovation Agency, Bogor, West Java, 16911, Indonesia

**Keywords:** Major mangrove, conservation, proboscis monkey, Penajam Paser Utara, IKN

## Abstract

**Background:**

*Camptostemon philippinensis*, found in mangrove forests in Indonesia and the Philippines, is listed as endangered on the International Union for Conservation of Nature (IUCN) Red List. It is found primarily in isolated mangrove forests in Kalimantan (Indonesian Borneo) and Sulawesi in Indonesia. Despite significant studies on mangrove biodiversity in this region, the occurrence of *C. philippinensis* in Balikpapan Bay, East Kalimantan, is not extensively recorded.

**Methods:**

The study was conducted by exploring the mangrove forests along Balikpapan Bay. The first survey of about 200 km was conducted to observe mangrove vegetation and found one *C. philippinensis* tree. The second survey focused on the area around the first discovered *C. philippinensis* tree to census and record its growth stage and distribution.

**Results:**

The study recorded a population of 527 individuals of *C. philippinensis* in Pantai Lango Village, East Kalimantan, dominated by seedlings. The high number of seedlings indicates good natural regeneration potential, but the low number of trees indicates intense competition for space in a restricted habitat. This species inhabits a small and restricted area in Balikpapan Bay, in the middle area of Balikpapan Bay, and is associated with other mangrove flora, such as *Rhizophora apiculata*, *Rhizophora mucronata*, *Sonneratia alba*, *Avicennia alba*, *Lumnitzera littorea*, *Osbornia octodonta*, *Ceriops tagal*, and *Xylocarpus granatum.*

**Conclusions:**

*C. philippinensis* is vulnerable to habitat damage from anthropogenic activities, which could lead to local extinction. Its natural habitat in Balikpapan Bay also has the potential to be under pressure due to the development of Indonesia’s new capital city ( *Ibu Kota Nusantara*/IKN). It emphasizes the need to understand the ecological role of this protected flora in the natural habitat of protected fauna (the proboscis monkey). Documenting the population of *C. philippinensis* is crucial for conservation efforts, including propagation and understanding its ecological role.

## Introduction

*Camptostemon philippinensis* (Vidal) Becc. is an endangered species comprising mangrove forests listed in the endangered category on the IUCN Red List ( Duke *et al.*, 2010). This species is one of Indonesia’s two protected mangrove species ( MEF Regulation Number 106/2018), so it is a high priority for conservation. *C. philippinensis* is a rare mangrove species globally distributed in Indonesia and the Philippines, with an estimated number of mature individuals of around 200 ( Duke *et al.*, 2010; Primavera *et al.*, 2004). In Indonesia, this species is only found on the islands of Kalimantan and Sulawesi ( Duke *et al.*, 2010) and mainly in isolated mangrove forests ( Djamaluddin, 2018). That is a lesser-known species, and so far, there are no records of its use by the community. However, the bark and leaves of *C. philippinensis* have the potential to contain anti-melanogenic and antibacterial chemicals ( Gadores *et al.*, 2021).

*Camptostemon philippinensis* is one of three species in the genus *Camptostemon* (Malvaceae) ( Beccari, 1886). This species was first published as *Cumingia philippinensis* S. Vidal in 1885, with type specimens originating from the island of Luzon. Beccarii (1886) suggested the move to the *Camptostemon* genus after observing the flower and fruit organs of the early species and discovering that the features in these two organs in *Cumingia* were comparable to those in *Camptostemon.* Van De Brink (1924) reduced the species *C. aruensis* to a synonym for *C. schultzii* based on his observations on the organs of flowers and fruits. That makes *C. philippinensis* and *C. schultzii* very easy to distinguish based on the morphological character of the leaf shape, where the first species has oblong leaf blades and the last species has elliptical leaf shapes. However, molecular studies have not been carried out specifically to support this opinion.

The population distribution of *C. philippinensis* on the island of Borneo is very limited. Previously, records of the distribution of this species in Borneo were only documented on the coast of Berau ( Mukhlisi & Sidiyasa, 2014). In Balikpapan Bay, several mangrove floristic studies have been conducted and recorded the richness of mangrove species, ranging from 12 species ( Kristiningrum *et al.*, 2019) to 20 species ( Warsidi & Endayani, 2017). Although various biodiversity studies have been carried out in the area, the presence of *C. philippinensis* has never been recorded ( Kristiningrum *et al.*, 2019; Sayektiningsih *et al.*, 2012, 2020; Toulec *et al.*, 2022; Willard *et al.*, 2022).

Balikpapan Bay is a mangrove ecosystem in East Kalimantan with an area of around 168 km ^2^ ( Toulec *et al.*, 2022), which is an essential habitat for various species of endangered wildlife, especially Borneo’s endemic primate, the proboscis monkey ( *Nasalis larvatus*). However, the threat of mangrove forest conversion is still present ( Anwar *et al.*, 2021b; Toulec *et al.*, 2022). The study results show that this area has one of the densest proboscis monkey population distributions, with as many as 60 groups ( Toulec *et al.*, 2022). On the other hand, mangrove vegetation has a strong interaction with the presence of proboscis monkeys because the monkeys like young leaves, seeds, and mangrove fruit as food sources ( Boonratana, 1993). Atmoko (2022) has reported as many as 41 plant species as food sources for proboscis monkeys from several habitats in East Kalimantan. In addition, proboscis monkeys often choose large mangrove trees on the banks of rivers or seas as sleeping trees to avoid predators. However, until now, information on the ecological role of *C. philippinensis* in the proboscis monkey’s natural habitats has been limited.

Documentation of the *C. philippinensis* population is important in supporting comprehensive conservation efforts, such as propagation efforts, and understanding its ecological role for the animals around it. More importantly, because some of the Balikpapan Bay areas will be developed as a site for the new Indonesian capital city ( *Ibu Kota Nusantara*/IKN), this study can be used as a resource for stakeholders interested in the usage of the area.

## Methods

A focused or intuitive-controlled survey ( Brewer, 2013) was conducted to observe *C. philippinensis.* This approach emphasizes surveying areas with high potential habitat for the target species, relying on the surveyor’s experience, field intuition, and readiness to steer the survey. We explored Balikpapan Bay to observe the mangrove vegetation in July 2022 and found a tree of *C. philippinensis.* The second survey in March 2023 focused on *C. philippinensis* existence. The survey was carried out using a boat along approximately 200 km of the coast of Balikpapan Bay. The study area covers Balikpapan City and Penajam Paser Utara Regency, where some will be part of the IKN Area. In the first survey, only one *C. philippinensis* tree was found on the shores of Kowangan Island, in the middle of Balikpapan Bay. In the second survey, we focused on the area where *C. philippinensis* had previously been discovered. We explored within a radius of 50 km from the point where the initial tree was found. We used the AvenzaMaps application on a smartphone to navigate and estimate distances in the field and used a Garmin CSx60 GPS receiver to take coordinates. The soil texture was observed *in-situ* following a guideline by Stuart-Street *et al.* (2020).

### Study area

The study area was in Balikpapan Bay, covering Balikpapan City and Penajam Paser Utara Regency, where some are part of the IKN Area (0°54′27″S-1°13′59S″ and 116°38′06″E-116°50′18″E). Balikpapan Bay is one of the largest remaining mangrove forests in East Kalimantan Province, with an area of 168 km ^2^ ( Toulec *et al.*, 2022). This bay is a semi-closed water body with 56 rivers and tributaries ( Kreb *et al.*, 2020). It holds high biodiversity value ( Lahjie *et al.*, 2019; Kreb *et al.*, 2020), including fisheries resources that have been utilized by local communities for more than 150 years ( Lahjie *et al.*, 2019). A permit to access and collecting C. philippinensis in Teluk Balikpapan was obtained from the Directorate General of Nature Resources and Ecosystem Conservation of the Ministry of Environment and Forestry of Indonesia, Number SK 94/KSDAE/SET.3/KSA.2/5/2023.

### Sample collection

A census was carried out by measuring the height and diameter of each *C. philippinensis* tree discovered in the survey area. We also recorded the coordinates of each individual and growth stage based on diameter at breast height (dbh), namely seedlings (germinated seed to <1.5 m in height), saplings (height >1.5 m; dbh <10 cm), and trees (dbh > 10 cm) ( Susilowati *et al.*, 2019) A brief description of the surrounding vegetation was carried out by recording the mangrove species assumed to be associated with *C. philippinensis.* The herbarium specimens of *C. philippinensis* were collected and deposited at Herbarium Wanariset (WAN) and Herbarium Bogoriense (BO).

Biogeographical information about the species was gathered from the herbarium specimens published on the GBIF website ( https://www.gbif.org/) ( GBIF, 2023). A literature review on the species, genus, or mangrove species that indicated the presence of *C. philippinensis* was also mined to enrich the distribution data and taxonomy information ( Noor *et al.*, 2012; Giesen *et al.*, 2006; Primavera *et al.*, 2004). All data and information were used to identify the distribution and describe the novel record of the species in Teluk Balikpapan, East Kalimantan.

### Data analysis

Each individual was counted to determine their total number based on growth stage (seedling, sapling, and tree) and is presented in the form of a percentage graph. Saplings and trees were evaluated for their average diameter and height to portray the population profile. This analysis was performed using Microsoft Excel 2016 (RRID:SCR_016137). Furthermore, the distribution of *C. phillipinensis* was mapped using Quantum GIS 3.30.

## Results

**Figure 1.**
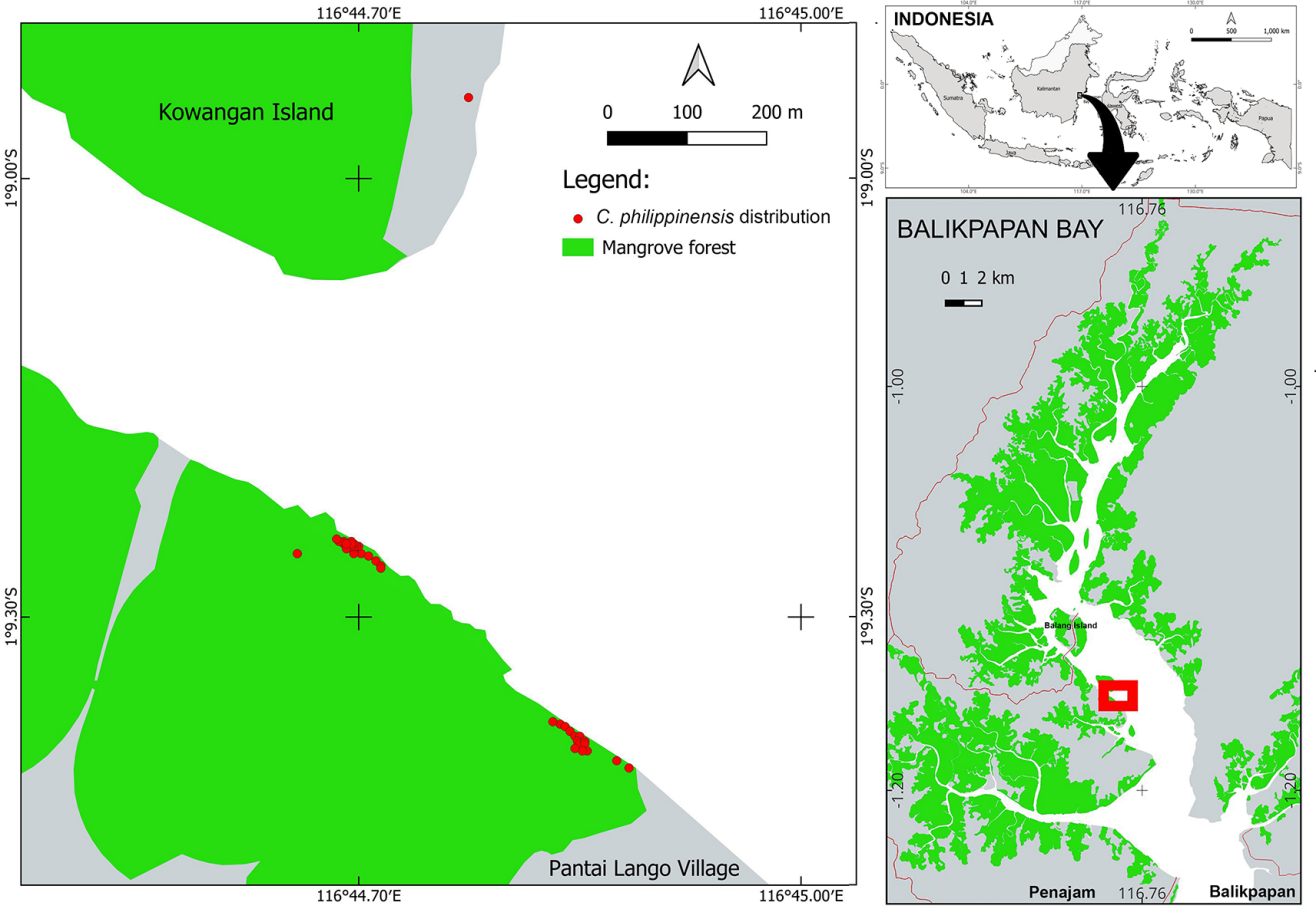
Habitat and distribution of *Camptostemon philippinensis* in Balikpapan Bay, East Kalimantan Indonesia.

*C. philippinensis* was discovered on Pantai Lango Village and Kowangan Island in Penajam Paser Utara District, East Kalimantan ( Figure 1) ( Mukhlisi, 2023; Sitepu *et al.*, 2023). It was discovered in a narrow area, although the Balikpapan Bay mangrove forest is relatively large, covering around 203 km ^2^ ( Toulec *et al.*, 2022). The *C. philippinensis* habitat is on the west shore, in the middle of Balikpapan Bay. Only one individual was found on a small island (Kowangan Island), whereas most others inhabited the mainland’s western shore in Pantai Lango. The distance between these two locations is around 500 m.

**Figure 2.**
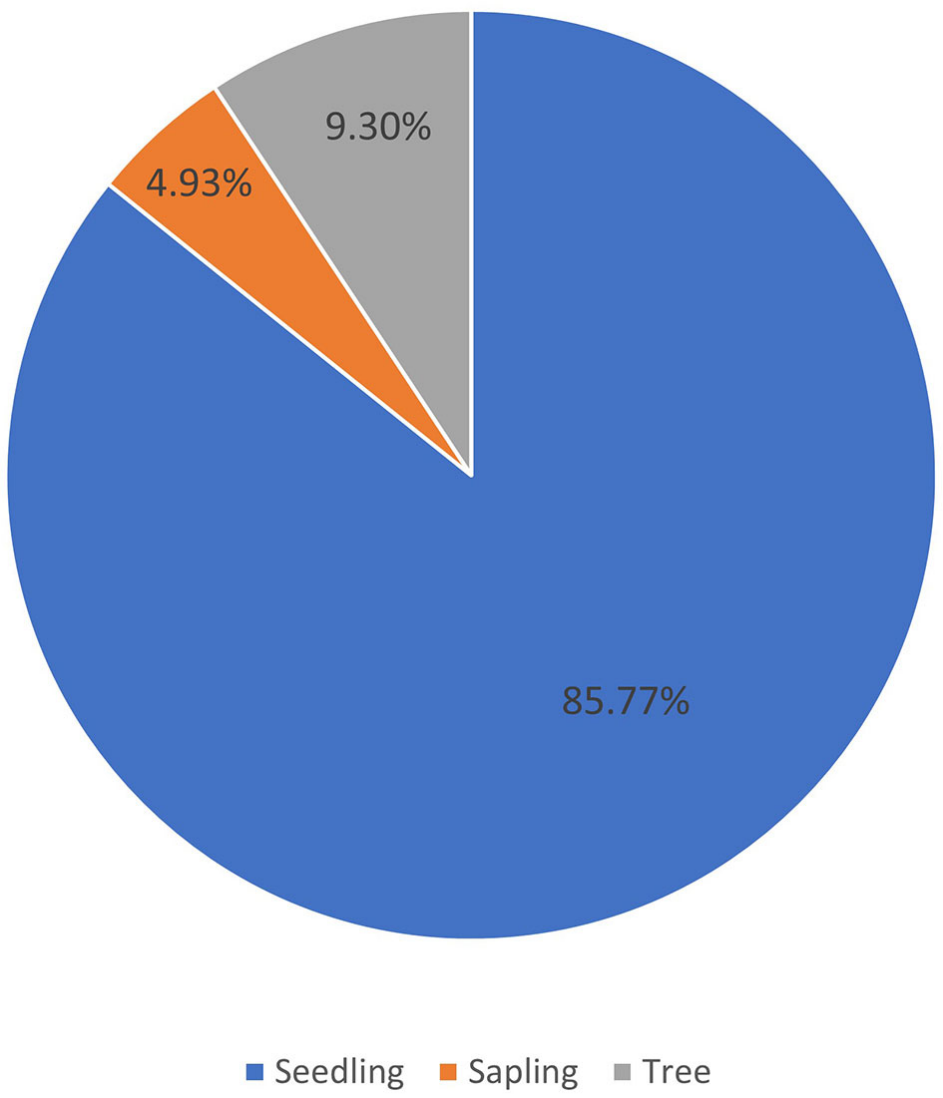
Percentage of the number of individuals of *Camptostemon philippinensis* in Pantai Lango based on the growth stage.

Based on our observations in Pantai Lango, there were around 527 individuals of *C. philippinensis* that were dominated by seedlings (452 individuals), followed by trees (49 individuals), and saplings (26 individuals) ( Figure 2). This demonstrates that even though *C. philippinensis* has a relatively limited range; its natural regeneration potential is functional. However, the number of saplings found was less than the tree stage, and this may be due to competition for space in a limited habitat. Generally, in mangrove forests, the number of seedlings is more abundant than the saplings, and the sapling stage is more numerous than the trees ( Islam *et al.*, 2015; Winata *et al.*, 2017).

**Figure 3.**
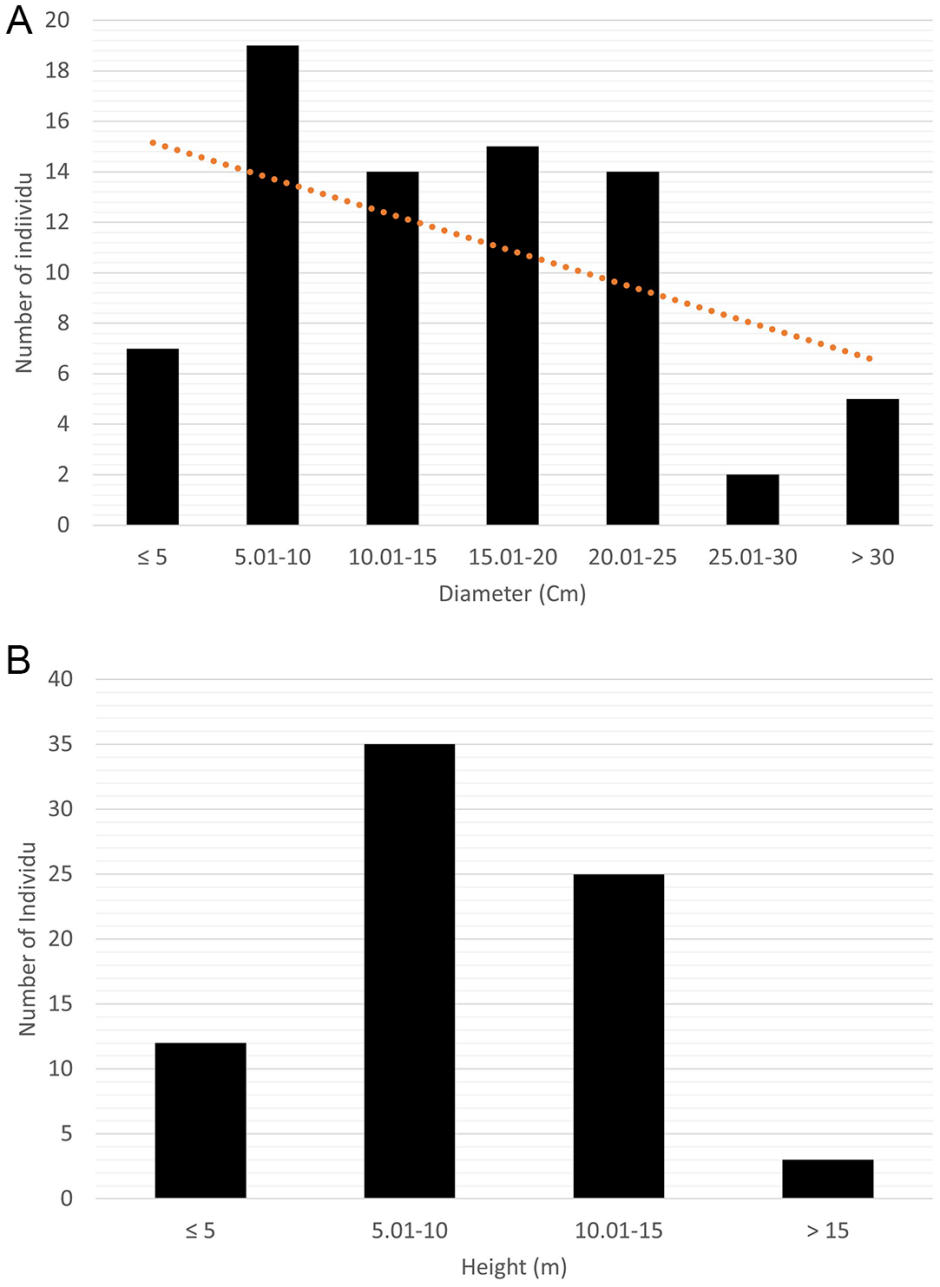
Profile of trees and saplings of *Camptostemon philippinensis* in Pantai Lango. A) Diameter and B) height.

**Figure 4.**
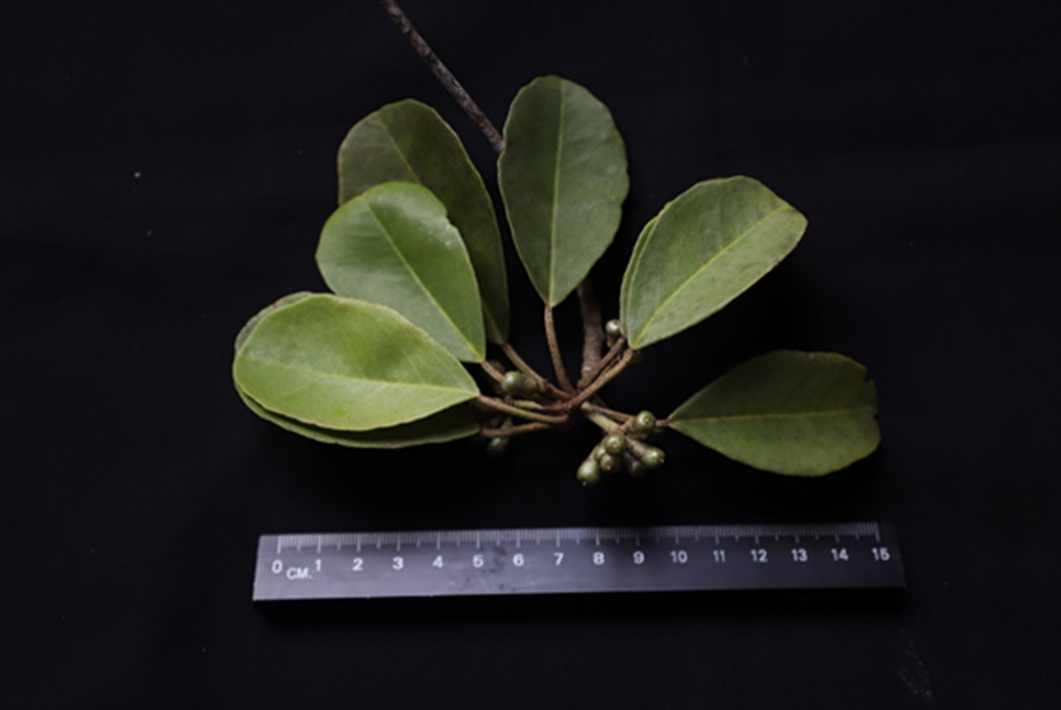
Fruiting specimens of *Camptostemon philippinensis* from Balikpapan Bay. The license mentioned in the Methods section included permission to collect specimens.

The saplings and trees of *C. philippinensis* have an average tree diameter of 14.98 ± 7.95 cm and an average height of 9.49 ± 3.59 m ( Figure 3). *C. philippinensis* in Balikpapan Bay has a diameter profile similar to the flora in the area. The tree was observed in the fruiting season in February 2023 ( Figure 4); however, no ripe fruits or flowers were observed.

A population of *C. philippinensis* was found in the middle area of Balikpapan Bay, and the influence of fresh water from river flow was not found significantly around its habitat. *In-situ* investigation showed that the soil texture around the habitat of *C. philippinensis* is predominantly sandy, both on the shore of the mainland (Pantai Lango) and on Kowangan Island. This species is frequently observed in environments impacted by moderate to low-intensity sea tides. Balikpapan Bay experiences daily semi-diurnal tides, with a tidal range of almost 2.5 m during spring tides and 1 m during neap tides ( Anwar *et al.*, 2021a). At the study site, *C. philippinensis* in the second layer of mangrove zonation, where the habitat does not flood during low tide. This differs from the vegetation in the first layer, which are observed to remain flooded even during low tide.

## Discussion

J. van Borssum Walkes identified *C. philippinensis* in Indonesia in 1962 based on herbarium specimens collected by CNJ Welmaar in 1919 from Pulau Laut, South Kalimantan ( Bakhuizen van den Brink, 1924; Troll, 1933) *.* In the following period, it was never recorded in various publications or scientific collections, so it was stated that it was no longer present in Kalimantan and grew only limitedly in the Papua region ( Macnae, 1968). However, Noor *et al.* (2012) continued to list this species along with *C. schultzii* as a type of mangrove plant in Kalimantan until publication by Mukhlisi and Sidiyasa (2014) based on botanical records in Tanjung Batu, Berau. While in Sulawesi, it was reported in Bunaken National Park ( Djamaluddin, 2018; Djamaluddin & Djabar, 2022) and Donggala ( Wahyuningsih *et al.*, 2012). In the Philippines, however, this species is reported to be widely spread but has yet to be abundant ( Primavera *et al.*, 2004).

In a limited habitat, populations of *C. philippinensis* tend to form stands in groups. The population is quite dominant at a certain point in a habitat, but not like *Rhizophora* spp. ( Mukhlisi & Sidiyasa, 2014). When compared with populations of other mangrove species in a wider sampling area, the population of *C. philippinensis* is classified as the lowest ( Djamaluddin, 2018; Pototan *et al.*, 2021; Wahyuningsih *et al.*, 2012). Several previous studies have also found a similar phenomenon where the population of *C. philippinensis* is known to be exclusive and limited to a narrow range of habitats ( Djamaluddin, 2018; Djamaluddin & Djabar, 2022; Mukhlisi & Sidiyasa, 2014). Although all mangrove propagules have buoyant abilities, which allow them to spread far through seawater ( Van der Stocken *et al.*, 2019), *C. philippinensis* requires specific ecological compatibility to colonize new habitats.

*C. philippinensis* often inhabits a small area; its dominant habitat in Balikpapan Bay is restricted to 300 m along the mainland shore. *Rhizophora apiculata*, *R. mucronata, Sonneratia alba*, *Avicennia alba*, *Lumnitzera littorea, Osbornia octodanta, Ceriops tagal*, and *Xylocarpus granatum* are among the mangrove flora associated with this species *.* The mangrove forest formation in this location has *C. philippinensis* in the second layer, behind the *R. apiculata*, *S. alba*, and *R. mucronata* formations. Meanwhile, one individual of *C. philippinensis* found on Kowangan Island inhabits the shoreline. On Kowangan Island, mangrove vegetation is situated next to a mountainous terrestrial forest in a narrow configuration that is often sparse. *Rhizophora apiculata*, *A. alba*, *Pongamia pinnata*, *Hibiscus tiliaceus, Lumnitzera littorea*, and *Osbornia octodonta* are associated with mangrove species on Kowangan Island.

We did not specifically observe the endangered species, proboscis monkeys (Nasalis larvatus). However, based on information from the local people (Darman, personal communication, 20 March 2022), he has seen two groups of proboscis monkeys on Kowangan Island and one other around the main island of C. philippinensis habitat. Our observations revealed primate bite scars on the leaves of *C. philippinensis.* This discovery is assumed to be proboscis monkey bite marks, given that leaves are the primate’s primary food source (folivorous) ( Boonratana, 1993). Other mangrove species on Balikpapan Bay reported to have a high preference as a food source for proboscis monkeys are *R. apiculata* ( Tangah & Bernard, 2010), *S. alba*, and *S. caseolaris* ( Atmoko, 2022).

Proboscis monkeys are arboreal primates that rely heavily on the presence of the mangrove tree canopy. They often use large and tall trees as sleeping sites at the water’s edge. The average diameter of *C. philippinensis* trees in Balikpapan Bay was similar to that in other locations ( Primavera *et al.*, 2004; Pototan *et al.*, 2021). In Banaybanay, Philippines, Pototan *et al.* (2021) found that the average dbh (diameter of breast height) of *C. philippinensis* was 16.25 cm. It can naturally reach heights of 15 m and diameters of up to 50 cm ( Primavera *et al.*, 2004).

**Figure 5.**
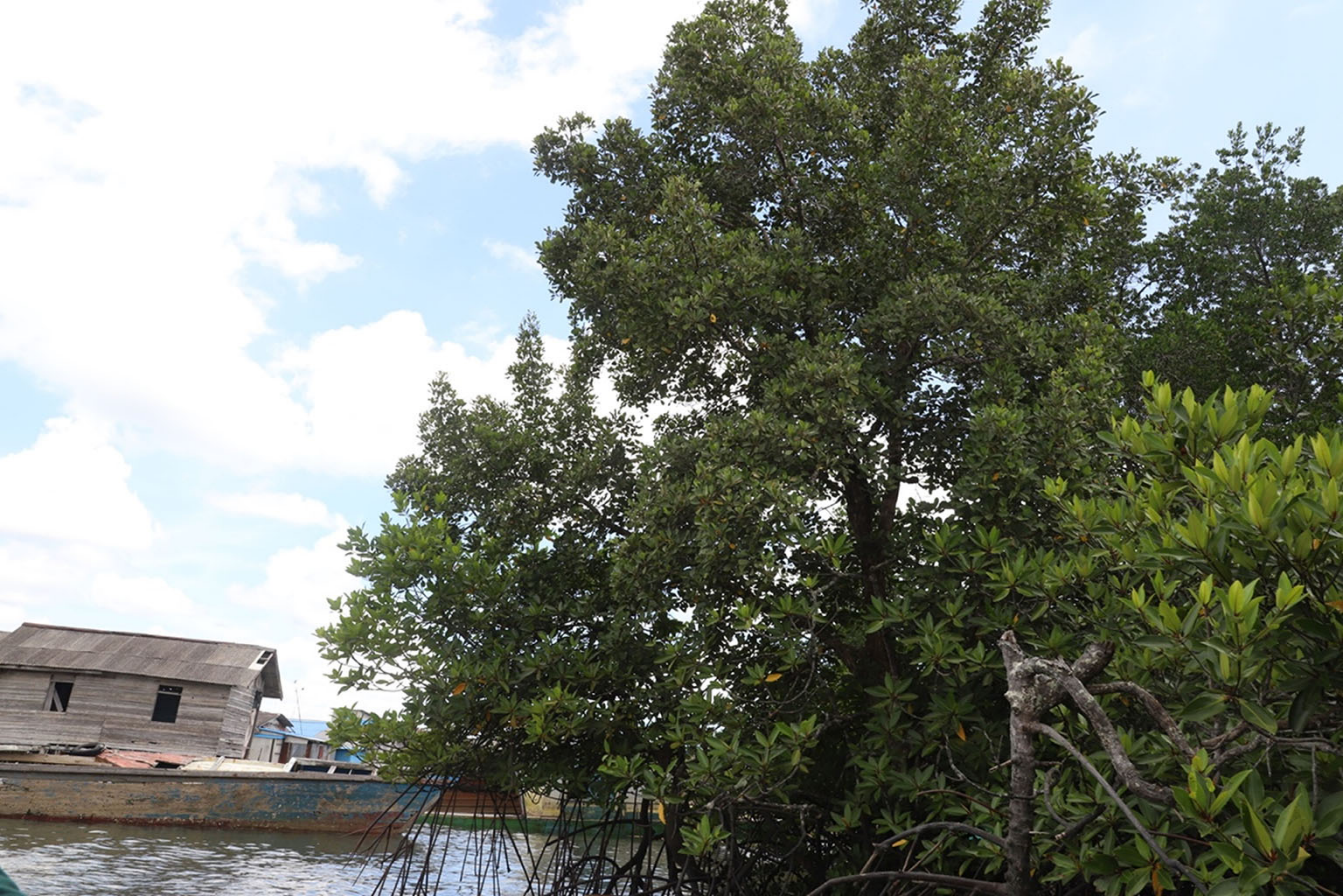
*Camptostemon philippinensis* tree in the shoreline of Pantai Lango close to the community settlement.

The threat of *C. philippinensis* populations in Balikpapan Bay is increasing, primarily due to anthropogenic activity. The population is localized in a narrow and extremely limited area; damaging its habitat increases the risk of local extinction ( Figure 5). Changes in the status of regions in the mangrove area of Balikpapan Bay and surrounding areas ( *e.g.*, the Indonesia’s New Capital City area (IKN)) would lead to alterations to the landscape and human population, which could pose a threat to its habitat. Current threats to *C. philippinensis* include the conversion of mangrove forests, environmental pollution, illegal logging, and the development of IKN.

Conserving *C. philippinensis* from extinction can be done with several efforts, such as prevention, species and habitat protection and preservation, habitat restoration, and *ex-situ* conservation. Prevention efforts can be carried out through education and outreach to various parties regarding the existence of *C. philippinensis* populations in the IKN area so that their habitat can be maintained. Protecting its natural habitat can be done by establishing a local protected area for the local community. Habitat restoration can be carried out by restoring degraded habitats and raising the number of individuals in their populations.

*C. philippinensis* in Indonesia has been listed as a protected plant under government law, and one of its natural habitats on Bunaken Islands has been designated as a conservation area and assigned as a national park. Unfortunately, no report on habitat restoration and *ex-situ* conservation efforts has been made until the present. Therefore, it is necessary to save genetic material from existing populations through seed storage and efforts to propagate both generatively and vegetatively, which can be used as a source of genetic material for habitat restoration or *ex-situ* conservation activities, as well as land rehabilitation. It is also necessary to study the genetic diversity of *C. philippinensis* in Indonesia, especially to determine conservation strategies, evaluate the success of restoration or reintroduction programs in other populations, and maintain genetic variation that will be important for the survival of this species.

## Conclusions

This study recorded 527 individuals of *C. philippinensis* found in Pantai Lango Village. This population has a complete structure, including seedlings, saplings, and trees at all levels. The natural regeneration potency of *C. philippinensis* is still functioning well, as indicated by the presence of many seedlings. However, intense competition for limited space in the habitat causes saplings to grow into the tree strata. It is under growing threat, primarily as a result of anthropogenic activities.

## Data Availability

Zenodo: All Raw Data of Camptostemon philippinensis in Balikpapan Bay, https://doi.org/10.5281/zenodo.8393832 ( Mukhlisi, 2023). Repositori Ilmiah Nasional (RIN) Dataverse: Data of Camptostemon philippinensis in Teluk Balikpapan, https://hdl.handle.net/20.500.12690/RIN/V8VTQV ( Sitepu *et al.*, 2023). Data are available under the terms of the Creative Commons Attribution 4.0 International license (CC-BY 4.0).
