# Supplementary material for: Camptostemon philippinensis, a new record of endangered mangrove species in the Balikpapan Bay, East Kalimantan, Indonesia
Source: F1000Res. 2025 Mar 31;12:1394. Originally published 2023 Oct 23. [Version 3] doi: 10.12688/f1000research.140887.3 (PMC12498319; doi:10.12688/f1000research.140887.3)
Supplement: Peer review history

# Reviewer report: Reviewer response for version 3

**DOI:** 10.5256/f1000research.178952.r374876

**Published:** 2025-05-09

**Authors:** Jhonnel P Villegas

**Affiliations:** 1 Davao Oriental State University, Mati City, Philippines

The study titled *Camptostemon philippinensis, a new record of endangered mangrove species in the Balikpapan Bay, East Kalimantan, Indonesia,* is generally well-written, and I recommend it for publication as a **Brief Report.** However, I would like to highlight several critical points for the improvement of the manuscript.

1. **Introduction**: The second part of the introduction requires revision. It discusses *C. aruensis*, a synonym of *C. schultzii*, which was not previously introduced, leading to incoherence in the narrative.
2. **Population Estimate**: The author reports documenting 572 individuals of *C. philippinensis*, but the methodology for obtaining this figure is unclear. The manuscript implies an absolute count of 572 individuals, although the actual number could vary. It is essential to include this in the methods section. While the researchers mention a 'census,' there is a need to elaborate on how this was conducted.
3. **Methodology**: The methods section should present the study area first, before all subsections. The term 'preliminary study' could be changed to 'survey' as it discusses the surveys conducted to document the mangrove species. There is also a need to elaborate on the process of identifying seedlings, saplings, and trees. The main identifying characteristics should be presented. Additionally, species identification confirmation by a mangrove taxonomist may be necessary, as this is a claim for a 'new record'.
4. **New Record Clarification**: It is worth noting that Balikpapan Bay has already been mapped in the global distribution range of *C. philippinensis *published in IUCN (Duke et al., 2008). Presenting this as a 'new record' could raise questions about the concept of new species records. It would be more appropriate to report this as the 'first' record in the bay, as this is still an important publication, considering the species is endangered. I request the editorial team to look into this more deeply. If this comment is deemed relevant, I suggest changing the title to " *Camptostemon philippinensis, the first record of an endangered mangrove species in the Balikpapan Bay, East Kalimantan, Indonesia."*
5. **Literature Review**: It is necessary to present existing literature (both gray and published), particularly those that studied mangrove diversity in Balikpapan Bay. This is crucial to demonstrate that extensive surveys have been conducted previously, but only now have the authors documented *C. philippinensis*.
6. **Discussion**: The discussion section may be enhanced to include information on how this discovery contributes to the global knowledge of the distribution of *C. philippinensis* and how it resonates with the current assessment of the IUCN Red List. Since the latest IUCN Red List assessment for this species was done in 2008, it is critical to briefly discuss how this discovery in the Balikpapan Bay could inform future assessments. The researchers could include recommendations to study the ecological relationships between *C. philippinensis* and *Nasalis larvatus*.
7. **Conclusion**: The conclusion needs enhancement, as it currently only summarizes what has been previously stated in the manuscript. There is a need to expound on the implications of the findings and provide useful recommendations for further research and conservation initiatives.

Is the work clearly and accurately presented and does it cite the current literature?

Yes

If applicable, is the statistical analysis and its interpretation appropriate?

Not applicable

Are all the source data underlying the results available to ensure full reproducibility?

No source data required

Is the study design appropriate and is the work technically sound?

Yes

Are the conclusions drawn adequately supported by the results?

No

Are sufficient details of methods and analysis provided to allow replication by others?

Partly

Reviewer Expertise:

wildlife ecology, conservation biology, and regenerative futures

I confirm that I have read this submission and believe that I have an appropriate level of expertise to confirm that it is of an acceptable scientific standard, however I have significant reservations, as outlined above.

# Reviewer report: Reviewer response for version 3

**DOI:** 10.5256/f1000research.178952.r374527

**Published:** 2025-05-08

**Authors:** Rene Rollon

**Affiliations:** 1 Institute of Environmental Science and Meteorology, College of Science, University of the Philippines Diliman, Quezon City, Metro Manila, Philippines

I appreciate the authors in addressing each of the general and specific points (mostly as annotations on the earlier version of the manuscript) raised in the previous review. I can now recommend approval of the paper, with the following reservations:

 (1) although I still disagree with using the term "new record", perhaps, using "local new record" (as also articulated in the reply) can be allowed. For this, I suggest to include this in the title, or at least, clearly reflected in the Abstract;

 (2)  on the re-focusing which I suggested earlier, the actions of the authors appeared substantial and can be acceptable;

 (3) on Fig. 3, I appreciate the revisions done by the authors, although the "break" on the y-axis which I suggested in my earlier review (see annotation on the manuscript) has not been implemented. I will attach a photo of such a suggestion here, and/or send through email if attachment with this review is not possible.

 All the best!

Is the work clearly and accurately presented and does it cite the current literature?

Yes

If applicable, is the statistical analysis and its interpretation appropriate?

Not applicable

Are all the source data underlying the results available to ensure full reproducibility?

No source data required

Is the study design appropriate and is the work technically sound?

Partly

Are the conclusions drawn adequately supported by the results?

Partly

Are sufficient details of methods and analysis provided to allow replication by others?

Yes

Reviewer Expertise:

Aquatic EcosystemsCoastal Marine Biology and EcologyMangrove and Seagrass EcologyCoastal Resources ManagementEnvironmental Impact AssessmentEnvironmental Sciences

I confirm that I have read this submission and believe that I have an appropriate level of expertise to confirm that it is of an acceptable scientific standard, however I have significant reservations, as outlined above.

# Reviewer report: Reviewer response for version 3

**DOI:** 10.5256/f1000research.178952.r374875

**Published:** 2025-04-22

**Authors:** Rahman Rahman

**Affiliations:** 1 Pattimura University, Ambon, Indonesia

This article provides valuable and timely local insight into the presence of a rare and endangered mangrove species, *Camptostemon philippinensis*, in Balikpapan Bay, East Kalimantan. The findings significantly contribute to the regional understanding of mangrove biodiversity and conservation priorities, especially in the context of growing anthropogenic pressures due to national development projects. The study is methodologically sound and well-documented, and with a few targeted improvements, it has the potential to meet a high scientific standard suitable for indexed.

 To strengthen the manuscript further, I recommend the following revisions:

1. **Expand the statistical treatment of demographic data**:
  - Include basic descriptive statistics (e.g., mean, standard deviation, and range) for key variables such as tree height and DBH (diameter at breast height).
  - Consider incorporating a simple analysis comparing the observed size-class distribution with expected patterns in naturally regenerating mangrove populations to provide ecological context to the findings.
2. **Clarify sampling limitations**:
  - Discuss the potential implications of the narrow sampling area (limited to approximately 300–500 meters) on the generalizability of the results across Balikpapan Bay.
  - Provide a brief justification for the selection of Pantai Lango as the primary study site over other potential locations within the bay.
3. **Enhance the vegetation context**:
  - Include a table or figure that summarizes the associated mangrove species observed alongside *C. philippinensis* in the study area.
  - If available, add information on the relative abundance or frequency of these species to enrich the ecological interpretation of the habitat.

By addressing these points, the manuscript will not only provide a clear and reliable record of *C. philippinensis* in a critical region but also offer a more comprehensive ecological narrative that will be valuable for conservation planning and biodiversity management.

Is the work clearly and accurately presented and does it cite the current literature?

Yes

If applicable, is the statistical analysis and its interpretation appropriate?

Partly

Are all the source data underlying the results available to ensure full reproducibility?

No source data required

Is the study design appropriate and is the work technically sound?

Yes

Are the conclusions drawn adequately supported by the results?

Yes

Are sufficient details of methods and analysis provided to allow replication by others?

Yes

Reviewer Expertise:

Mangrove ecology and its biodiversity, mangrove blue carbon management, mangrove conservation.

I confirm that I have read this submission and believe that I have an appropriate level of expertise to confirm that it is of an acceptable scientific standard, however I have significant reservations, as outlined above.

# Reviewer report: Reviewer response for version 3

**DOI:** 10.5256/f1000research.178952.r374528

**Published:** 2025-04-02

**Authors:** Inocencio E Buot

**Affiliations:** 1 Institute of Biological Sciences, College of Arts and Sciences, University of the Philippines Los Banos, Laguna, Calabarzon, Philippines

I appreciate the responses of the authors to my comments. Most of these are alright. I have reservations however, of the phrase "Preliminary study" under Methods?

 By preliminary, you mean this was done before the actual field measurements described under "Sample Collection"? 

 Will "preliminary study be the appropriate subsection title?

 I will this part to the editor.

 All my other comments had been answered well. I am approving this paper now.

Is the work clearly and accurately presented and does it cite the current literature?

Yes

If applicable, is the statistical analysis and its interpretation appropriate?

No

Are all the source data underlying the results available to ensure full reproducibility?

Partly

Is the study design appropriate and is the work technically sound?

Yes

Are the conclusions drawn adequately supported by the results?

Yes

Are sufficient details of methods and analysis provided to allow replication by others?

Partly

Reviewer Expertise:

Botany (ecology and systematics)

I confirm that I have read this submission and believe that I have an appropriate level of expertise to confirm that it is of an acceptable scientific standard.

# Reviewer report: Reviewer response for version 2

**DOI:** 10.5256/f1000research.159723.r263530

**Published:** 2024-05-31

**Authors:** Rene Rollon

**Affiliations:** 1 Institute of Environmental Science and Meteorology, College of Science, University of the Philippines Diliman, Quezon City, Metro Manila, Philippines

This paper reports on the occurrence of mangrove species *Camptostemon philippinensis*, supposedly a new record in Balikpapan Bay, East Kalimantan, Indonesia. The authors found 527 individuals of the species in one location (Pantai Lango), while only one individual in another (i.e., Kowangan Island, some 500 m away from the former). Of these individuals, seedlings comprised >85% (452), with saplings (4.9%; 26) and trees (9.3%; 49) together accounting for less than 15%. Such demographic profile may be indicative of high mortality rate from seedling - (85.8%) to sapling (4.9%) growth stage, and hence, may be an alarm button for further understanding better its population dynamics, hopefully leading to design concrete management actions to conserve this endangered species in the area.

**General comments:**

 Although the paper, as it is now, indeed appears to be relatively well-written, I found several issues that in my opinion should be resolved before the paper may be considered for indexing:

1. “ **new record**”: this paper is reporting the occurrence of *Camptostemon philippinensis* in East Kalimantan, which may indeed be a “new record” for the locality, but not really unexpected (and therefore not of news value) considering that Balikpapan Bay (East Kalimantan) is actually part of the published extant range of this species (see Duke *et al.*, 2010). What would be more interesting to report as “new records” are those occurrences outside the published range. Otherwise, finding 1,000 local “new records” within the known range of the species would result in 1,000 “new record” publications on the species?
  In fact, the occurrence of this species far more south of East Kalimantan coast has already been reported (i.e., Pulau Laut; Walkes 1962 based on herbarium specimens collected more than a century ago [1919]) and as also reflected in Fig. 1 (top chart) of this manuscript. Because Balikpapan Bay is located between Pulau Laut and Berau (where occurrence of this species has also been recorded), and also because this bay is located close to the west coast of Sulawesi where this species is also present (Fig. 1, top panel), it should not be surprising to find pocket stands (e.g., Balikpapan Bay) of this species within the range previously established. In my opinion, this finding is not unexpected, and therefore of little news value to readers.
2. **Re-focus**: I would rather like to suggest to re-focus the manuscript from its current “new record” narrative into something like demographic profile of *Camptostemon philippinensis* near its southeastern global distribution limit. For such a reorientation, Fig. 3 would be a critical part of this new narrative. I have scribbled (annotated) as to how this figure could be made more intuitive, but perhaps a lot more information may be needed. For instance, more comparative approaches (e.g., Balikpapan vs. Berau vs. Sulawesi) and then use statistical tests for these comparisons rather than completely absent as it is now.
3. **Figure 3**: This figure may be improved immensely. Chart A can be interesting, but as it is now, it does not appear intuitive. The Chart is actually showing size-frequency distribution of the species which theoretically, should follow an exponential decay curve. I tried to combine your values (see annotations) and indeed, the Chart can look better, approximating more closely the natural (expected) trend. Chart B as it is now is not really relevant in the “narrative” and may be deleted.

**Specific comments:**

 I also have several specific comments as annotations on the manuscript - please see the PDF file linked here.

 All the best!

Is the work clearly and accurately presented and does it cite the current literature?

Yes

If applicable, is the statistical analysis and its interpretation appropriate?

Not applicable

Are all the source data underlying the results available to ensure full reproducibility?

No source data required

Is the study design appropriate and is the work technically sound?

Partly

Are the conclusions drawn adequately supported by the results?

Partly

Are sufficient details of methods and analysis provided to allow replication by others?

Yes

Reviewer Expertise:

aquatic ecosystems; coastal marine biology and ecology; mangrove- and seagrass ecology; coastal resource management; environmental impact assessment;  environmental sciences

I confirm that I have read this submission and believe that I have an appropriate level of expertise to state that I do not consider it to be of an acceptable scientific standard, for reasons outlined above.

# Response

**Published:** 2025-03-04

**Authors:** Abdul Razaq Chasani

**Affiliations:** Biology, Universitas Gadjah Mada, Yogyakarta, Special Region of Yogyakarta, Indonesia

We appreciate your willingness to review the publication and provide valuable advice and we accept the inputs with some improvement in the paper

 Respond to general review points 1-3.

1. We agree that this publication uses the term " **new record**" for the local new record; however, we will maintain the term to emphasize that this is the first time C. philiphinensis scientifically reported from Balikpapan Bay, when previous studies in a similar area have not recorded this species (Warsidi & Endayani, 2017, Kristiningrum et al., 2019; Sayektiningsih et al., 2012, 2020; Toulec et al., 2022; Willard et al., 2022). It should be noted that Balikpapan Bay is a very large mangrove ecosystem reaching 168 km2 that is facing the threat of ecosystem change due to the construction of Indonesia’s new capital city. The term is also used to attract stakeholders and or policymakers to pay attention to and maintain the existence of this population and their habitat. In this publication, we also add suggestions regarding conservation strategies for this species.
2. We accept reviewer feedback by making changes on demographic aspects.
3. We improve figure 3 and add an explanation for it based on reviewer recommendations. We removed Fig. 3. B. and revised the DBH categories into 4 groups (<5 cm; 5-15; 15-25 and >25)

Detail of response from the reviewer comment attached in the PDF linked here. Thank you for your comment and review, we improved the article based on the reviewer comments.

**Competing Interests:** No competing interests were disclosed.

# Reviewer report: Reviewer response for version 2

**DOI:** 10.5256/f1000research.159723.r237864

**Published:** 2024-02-23

**Authors:** Inocencio E Buot

**Affiliations:** 1 Institute of Biological Sciences, College of Arts and Sciences, University of the Philippines Los Banos, Laguna, Calabarzon, Philippines

I am approving the latest revision of the manuscript.

Is the work clearly and accurately presented and does it cite the current literature?

Yes

If applicable, is the statistical analysis and its interpretation appropriate?

No

Are all the source data underlying the results available to ensure full reproducibility?

Partly

Is the study design appropriate and is the work technically sound?

Yes

Are the conclusions drawn adequately supported by the results?

Yes

Are sufficient details of methods and analysis provided to allow replication by others?

Partly

Reviewer Expertise:

Botany (ecology and systematics)

I confirm that I have read this submission and believe that I have an appropriate level of expertise to confirm that it is of an acceptable scientific standard.

# Reviewer report: Reviewer response for version 1

**DOI:** 10.5256/f1000research.154292.r218353

**Published:** 2023-11-10

**Authors:** Inocencio E Buot

**Affiliations:** 1 Institute of Biological Sciences, College of Arts and Sciences, University of the Philippines Los Banos, Laguna, Calabarzon, Philippines

General comments

1. The paper is very well written actually.
2. This is a good report as new occurrence record, but I am suggesting some more information  if possible.
3. The first paragraph under methods should have a subsection title since all the rest that follows have. Would that be reconnaissance perhaps?
4. I think Study Area should come first as a subsection under Methods so that the authors can discuss why focus in that mangrove forest when there are so many in Indonesia? This discussion is lacking under Study Area. Were there clues or hints that they could spot the species in the area? Were the first herbarium documentations playing a key role in leading them to the area of investigation?
5. The herbarium specimens used for biogeographic studies were not tabulated with details of the exsiccata. I would suggest having that to add veracity of the information. And even all the vouchers examined and mentioned in the results and discussions.
6. There was no statement in the method on the use of drone to start of the reconnaissance survey. That could have been resorted to first, before ground truthing and spotting the tree using a boat. But so long as there was a thorough ground truthing anyway, that would be fine.
7. In the results, I expect the details of the exsiccata of the herbarium vouchers collected by the authors from the study area and deposited at Herbarium Wanariset (WAN) and Herbarium Bogoriense (BO). If voucher photographs are available, it would be great to see one here in this paper. And of course, photos of the plant are wanting with details of the different organs preferably with measurements, so that readers would not have reservations of its identity.
8. Fig 1 seems to include only field data? Would you not consider including here the distribution record of the species as documented in the herbarium collections in the past as mentioned in the discussion portion of the paper?
9. In the discussion, there was no mention of the currently gathered data except for the reference to Fig. 5. Hence, it would appear a review paper since the data collected were not cited well.

I commend the authors for doing this study and writing the paper. I hope to see this revised and published soon.

Is the work clearly and accurately presented and does it cite the current literature?

Yes

If applicable, is the statistical analysis and its interpretation appropriate?

No

Are all the source data underlying the results available to ensure full reproducibility?

Partly

Is the study design appropriate and is the work technically sound?

Yes

Are the conclusions drawn adequately supported by the results?

Yes

Are sufficient details of methods and analysis provided to allow replication by others?

Partly

Reviewer Expertise:

Botany (ecology and systematics)

I confirm that I have read this submission and believe that I have an appropriate level of expertise to confirm that it is of an acceptable scientific standard, however I have significant reservations, as outlined above.

# Response

**Published:** 2023-12-04

**Authors:** Abdul Razaq Chasani

**Affiliations:** Biology, Universitas Gadjah Mada, Yogyakarta, Special Region of Yogyakarta, Indonesia

1. Thank you for providing a review and comments on this paper.

 2. We added more information as suggested.

 3. A subsection title (preliminary survey) was added under the methods title.

 4. The Study Area subsection moved to the first subsection, followed by the Preliminary Survey subsection.

 The study area selection has been discussed in the methods and discussion section. The study area is an essential ecosystem located in an area that could be affected by the development of Indonesia's new capital city, so the general mangrove survey is important. A single Camptostemon philippinensis tree was spotted during the preliminary survey, collected as an herbarium, and confirmed as C. philippinensis. A second survey was then conducted to find other C. philippinensis trees in the nearby area.

 5. We added the exsiccate and the figure of the herbarium. We also added a map and a table to list the herbarium collection from Indonesia.

 6. We did not use a drone for the survey; all of the surveys were conducted on a boat, traveling from upstream to downstream and back.

 7. We added the figures of the herbarium deposited in BO and provided the figures of the flower (Fig. 4b) and fruit (Fig. 4c).

 8. We have revised Figure 1 and added the distribution record of the species.Capmtostemon philipinensis

 9. The present study has described the data collection in paragraphs 2 and 3.

 10. We have revised our manuscript and provided more information as reviewer suggestions.
